# A Review on Scorpionism in Iran

**Published:** 2018-12-25

**Authors:** Rouhullah Dehghani, Esmail Charkhloo, Nedasadat Seyyedi-Bidgoli, Elahe Chimehi, Maedeh Ghavami-Ghameshlo

**Affiliations:** 1 Department of Environmental Health Engineering, Social Determinants of Health Research Center, Kashan University of Medical Sciences, Kashan, Iran; 2 Department of Environmental Health Engineering, Faculty of Health, Jiroft University of Medical Sciences, Jiroft, Iran

**Keywords:** Province, Sting agent, Scorpion, Iran

## Abstract

**Background:**

Scorpions are one of the most important venomous animals in Iran. Their sting has more prevalence in the south and southwest areas. The aim of this study was to introduce their sting agent species in the country.

**Methods:**

Data were extracted by a mini review on scorpion stinging articles in Iran until early 2018 and then the sting agent species in each area were studied. Geographical and provincial distribution of each species also was provided.

**Results:**

Twelve scorpion species are causative agents of sting. According to their deadly rate and clinical symptoms, some of them are considered the most dangerous venomous animals in Iran. Some death cases have been reported because of the sting of 3 species of *Hemiscorpius lepturus, H. acanthocercus* and *Androctonus crassicauda*. Remaining species have not deadly sting but because of their frequency, they encounter the individuals and cause the stinging.

**Conclusion:**

The highest number of sting agents is in Khuzestan, however Gilan and Mazandaran have the lowest frequency. Because of the high prevalence of sting agent species in that province, the necessity of providing control and prevention programs is very important.

## Introduction

Scorpion stinging has been reported in most of the warm areas of the world. These animals belong to the Arthropoda. Scorpion’s geographic distribution is all over the world and the latitude between equator North 50 degree and south 52 degree (1) but even in this geographic area, they have different distribution. These animals have been seen in many habitats and are able to live in very tough conditions. They use the least energy (2). They sting in order to defend and feed. They are opportunist in terms of selecting their habitat and using any natural and artificial or human-made spaces and gaps for hiding and habitat (3, 4). Some of them are nest makers and diggers. They make nests in the soil with smother soil pattern and proper physical structure (5–7). Some species of these animals have adapted their activity inside or around the human residential areas and for this reason their probable encounter with humans has increased. Therefore, in these cases, sting threat is more in comparison with active species which are out of and farther from the human residential places (8–11).

In Iran, the species which sting humans are more opportunist in terms of habitat selection. They use ready spaces and gaps provided in the buildings because of using traditional building materials. This arthropod starts its activities at night and uses its venomous sting to defend or hunt insects to feed. The habitat of most of them is desert and non-residential places (12–14). Since these animals are hunters, like tarantulas, this kind of habitats attract the animals which are these hunters’ food. On the other hand, the scorpion hunters are attracted and make a complete food web with different food chains. Therefore, this kind of places, in addition to providing proper shelter and habitat, make their food available (15–17).

Scorpions are dangerous for humans because of having toxic and deadly sting and for this reason they are medically important so that according to the available statistics, they have the highest human casualties by venomous arthropod in the world (8, 18). Till now, 64 scorpion species have been reported in Iran (2) but no report is available if they all sting or not. The population frequency of scorpion species in Iran is more than other stinging and biting animals like venomous and non-venomous snakes; therefore, their consequence is more stings (19, 20). In Iran, scorpion sting is about 10 times more than snake biting. The highest human fatalities are caused by venomous arthropods in the world (21–23). Scorpion sting which threatens many people to death annually is one of the most important health issues in tropical and subtropical areas like Iran. The highest statistics of stings and fatalities belong to Khuzestan and Hormozgan (24–30).

Since many species live in Iran but sting of all of them has not been reported, the purpose of this study is introducing the sting agent species and determining their province distribution during the past 50 years in Iran.

## Materials and Methods

In this review article, keywords like scorpion, sting agents, dangerous species, provincial distribution, Iran, identification, studies, family and species were used in the sites related to valid medical and health journals, searching in databases like Web of Science, Ovid, PubMed, Systematic Review, SID, Iran Medex, Scirus, Google Scholar and Medline to have access to the articles during 1977 until early 2018. The including criteria for entry in this study articles were as follows, the first all Iranian articles about animal bites were searched. In the next step, they study focused on venomous animal bites and stings. Then the sting agents among of the scorpions were noticed, and then all articles of the scorpion sting agents in the past decades till now have been noticed. Overall, 150 sources were found, but only 75 of these considering the purpose of the study; i.e., report of the sting agent and concentration of study on Iran, 73 sources were surveyed. In addition to the survey of these studies, their application in Iran was done. Then the gained results were provided in tables, graph and figure.

## Results

Up to now, three scorpion families have been reported in Iran. The sting agent scorpions in Iran include two families of Buthidae and Hemiscorpiidae. They have 12 species from the 8 genus which 10 species belong to Buthidae family and 2 species belong to Hemiscorpiidae family. More than 83.5% of the identified sting agent species in Iran belong to Buthidae family and 16.5% belong to Hemiscorpiidae family (29, 2, 31, 32, 33). Identification of sting agent species among the scorpions of Iran has been done by different researchers especially in the field of medical sciences. Still, there are changes in the number of families, genus and species of sting agents in Iran so that in the initial reports of researchers, sting agent scorpions in Iran were introduced to be 3 to 4 species while they are 12 species now (2, 31). According to the last studies about scorpion sting agent species, there are 2 families in Iran: Buthidae and Hemiscorpiidae.

**Fig. 1.**
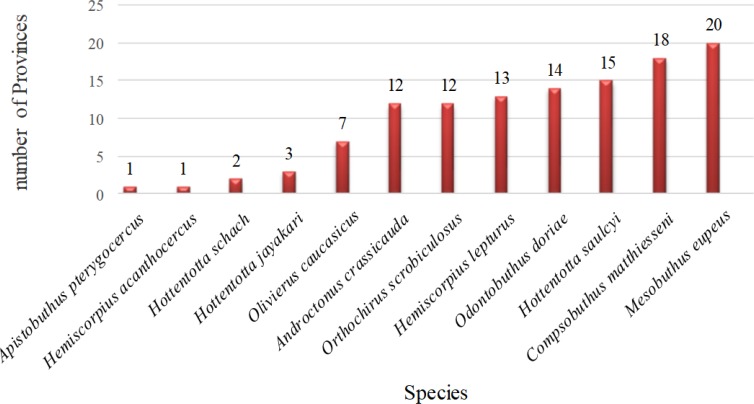
Provincial abundance of scorpion sting agents in Iran

The species of *Mesobuthus eupeus* are in Ardabil, Kerman, Isfahan, Markazi, Mazandaran, Sistan and Baluchistan, Yazd, Kohgiluyeh and Boyer-Ahmad, Semnan, Fars, Khuzestan, Hormozgan, Golestan, Tehran, Kordistan, Kermanshah, Ilam, west Azarbaijan, Khorasan Razavi and Khorasan Jonoobi. Then, *Compsobuthus matthiesseni* in Bushehr, Chaharmahal and Bakhtiari, Fars, Hamadan, Kerman, Kohgiluyeh and Boyer-Ahmad, Kordistan, Lorestan, Markazi, Qom, Khuzestan, Hormozgan, Khorasan, Kermanshah, Ilam, west Azarbaijan and Isfahan, *Hottentotta saulcyi* in Lorestan, Hamadan, Chaharmahal and Bakhtiari, Khuzestan, west Azarbaijan, Kermanshah, Hormozgan, Ilam, Sistan and Balochistan, Kordestan, Kohgiluyeh and Boyer-Ahmad, Fars, Isfahan, Kerman and Ardabil, *Odontobuthus doriae* in Hormozgan, Kerman, Yazd, Isfahan, Markazi, Ghazvin, Tehran, Alborz, Semnan, west Azerbaijan, Kermanshah, Busher, Hamedan, Hormozgan, *Hemiscorpius lepturus* in Khuzestan, Semnan, Fars, Kordestan, Hormozgan, Bushehr, Ilam, Lorestan, Kermanshah, Isfahan, Hamedan, Kohgiluyeh and Boyer-Ahmad and Kerman, *Orthochirus scrobiculosus* in Khuzestan, Hormozgan, Tehran, Sistan and Balochestan, Qom, Isfahan, median (Razavi) Khorasan, Khorasan Jonobi, Gilan, Semnan, Kermanshah, Ilam, *Androctonus crassicauda* in Bushehr, Semnan, Khuzestan, Ilam, west Azarbaijan, Kordestan, Khorasan Razavi, south Khorasan, Kermanshah, Kerman and Sistan and Balochistan, *Mesobuthus* or *Olivierus caucasicus* in west Azarbaijan, Sistan and Balochestan, Isfahan, south Khorasan, Tehran, Markazi and Semnan, *Hottentotta jayakari* in Qom, Hormozgan and Fars, *Hottentotta schach* in Fars and Khuzestan, *Hemiscorpius acanthocercus* in Hormozgan and *Apistobuthus pterygocercus* in Khuzestan have been reported, respectively (31–53) (Table 1, Fig. 1).

**Table 1.** Scorpion sting agent in Iran based on family, genus and species

**Family**	**Genus**	**Species**	**Number of provinces**	**Author**
**Buthidae**	Mesobuthus	*Mesobuthus eupeus* (C. L. Koch,1839)	20	2, 30, 29, 28, 24, 23
**Buthidae**	Compsobuthus	*Compsobuthus matthiesseni* (Birula, 1905)	18	8, 13, 14, 2, 16
**Buthidae**	Hottentotta	*Hottentotta saulcyi* (Simon, 1880)	15	8, 18, 23–28
**Buthidae**	Odontobuthus	*Odontobuthus doriae* (Thorell, 1876)	14	8, 23, 25, 31, 32, 33
**Hemiscorpiidae**	Hemiscorpius	*Hemiscorpius lepturus* (Peters, 1862)	13	34–51
**Buthidae**	Orthochirus	*Orthochirus scrobiculosus* (Birula, 1900)	12	8, 18
**Buthidae**	Androctonus	*Androctonus crassicauda* (Olivier, 1807)	12	8, 18, 28, 27, 30, 52
**Buthidae**	Mesobuthus or Olivierus	*Mesobuthus or Olivierus caucasicus* (Nordmann, 1840)	7	8, 18
**Buthidae**	Hottentotta	*Hottentotta jayakari* (Pocock, 1895)	3	28
**Buthidae**	Hottentotta	*Hottentotta schach* (Birula, 1905)	2	8, 18
**Hemiscorpiidae**	Hemiscorpius	*Hemiscorpius acanthocercus* (Monod et Lourenço, 2005)	1	53
**Buthidae**	Apistobuthus	*Apistobuthus pterygocercus* (Finnegan,1932)	1	8, 18
**2**	8	12		

All the provinces of Iran have at least one or some species of scorpions of Scorpionida order which cause the sting. In Iran, the number of sting agents of order Scorpionida in Fars Province is 12 species. Overall, 64 species of scorpions have been reported in Iran so far (2, 29). *Mesobuthus eupeus* has a wide geographic distribution in Iran and at least has been reported in 20 provinces. Most of the sting agent species are found in the South of Iran specially Khuzestan. During the last years, about 80% of all the reported sting cases in Iran have been from this area. The sting of *H. jayakari* has been first reported from Iran (8, 18).

**Fig. 2.**
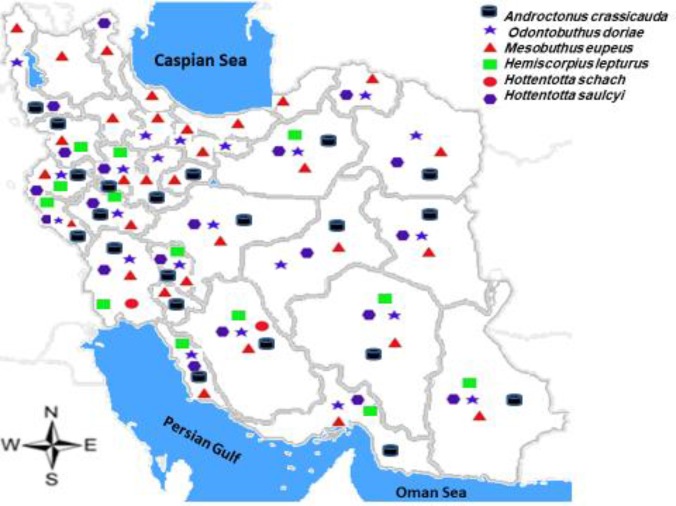
Species provincial distribution of major scorpion sting agents in Iran

According to the reports, scorpions’ venoms which their LD_50_ is measured less than 1.5mg/kg, in mice are considered to be in the dangerous and or deadly group. Among the species in Iran, LD_50_ of 5 species of this arthropod has been measured among which *M. eupeus* is in the dead border and has been measured as 1.45mg but LD_50_ of others is less (Table 2). LD_50_ of *H. lepturus* is much more than Buthidae family species but because of the delay mechanism of the venom of this arthropod, it is considered as one of the most deadly species in Iran (8, 18, 33, 54, 55). The highest geographical distribution of sting agent scorpions is related to *M. eupeus* and *C. matthiesseni* reported in 20 and 18 provinces, respectively. In this study, the minimum geographical distribution belongs to *H. acanthocercus* reported from Hormozgan and *A. pterygocercus* reported from Khuzestan (8, 18, 21). *Apistobuthus pterygocercus* is described as *A. susanae* based on new samples in Khuzestan Province. However, in the reports, the name is *A. pterygocercus* as a stinging agent in Khuzestan (8, 18, 56) (Fig. 2).

**Table 2.** LD_50_ of sting agent scorpions in Iran based on the injection method

**Species**	LD_50_*	Method**	**Family**
***Androctonus crassicauda***	0.08–0.50	Sc/iv	Buthidae
***Odontobuthus doriae***	0.19	iv	Buthidae
***Hottentotta saulcyi***	1.01	iv	Buthidae
***Mesobuthus eupeus***	1.45	iv	Buthidae
***Hottentotta schach***	3.36–4.2	iv	Buthidae
***Hemiscorpius lepturus***	5.81	iv	Hemiscorpidae

* The dose is expressed in mg of venom per kg of mouse

** Method: iv= intravenous injection, ip= intraperitoneal injection, sc= subcutaneous injection

## Discussion

The number of scorpion species has been rapidly increasing in the last 3 decades in the world (57–61). The number of described species in the world has reached 2231 classified in 208 genus and 20 families. The family Buthidae with a higher frequency than others are scattered all over the world. This family includes the most dangerous species. From this family, deadly species live in Iran. In addition, the family Hemiscorpiidae from *Hemiscorpius* genus includes dangerous and deadly species in the Middle East especially Iran and Iraq and are classified as the most deadly scorpions of the world (62–71). At present, one of the control methods of health is the use of pesticides, that it may cause resistance to several of urban pests such as scorpions, flies and etc., therefore, the improvement of the environment and the removal of shelters could reduce the risk of scorpion stings (72–75).

## Conclusion

At present, the most dangerous species of scorpions are in the South and Southwest of Iran. However, completing the data about the sting agent scorpions’ species in Iran needs more efforts of young researchers. Meanwhile, the completion of data in the field of Iran’s sting agent scorpion species and different aspects of it needs cooperation between the physicians of the venomous animal’s sting therapy units and the entomologist in this field. More accurate studies will be done with the cooperation of specialists of different fields about the sting agent species and the clinical effects of each species. This work necessitates a complete research in the country with a similar method and in the provinces and cities. Still, the highest species diversity is seen in the South and Southwest provinces but the diversity of scorpions in the Northeast and Northwest of Iran is less than the Southwest. In high-risk cities and villages, we recommend to the authorities of emergency department of hospitals and treatment centers, to emphasis on having scorpion sting agent by victim companions, because it helps to accurately identify sting agent.
